# No one-to-one mapping between typologies of pragmatic relations and models of pragmatic processing: a case study with mentalizing

**DOI:** 10.1098/rstb.2023.0501

**Published:** 2025-08-14

**Authors:** Napoleon Katsos, Mikhail Kissine

**Affiliations:** ^1^ Section of Theoretical and Applied Linguistics, Trinity College, University of Cambridge, Cambridge, UK; ^2^ ULB Neuroscience Institute, LaDisco, ACTE, Université libre de Bruxelles, Bruxelles, Belgium; ^3^ Department of Philosophy, Classics, History of Art and Ideas, University of Oslo, Oslo, Norway; ^4^ Department of Linguistics and Comparative Cultural Studies, Ca' Foscari University of Venice, Venice, Italy

**Keywords:** pragmatics, implicature, irony, autism

## Abstract

In this article, we argue that the growth of research in cognitively and experimentally oriented pragmatics in the last two decades has rested on two epistemological assumptions: that theoretical-pragmatic notions such as ‘implicature’, ‘metaphor’ and ‘irony’ correspond to distinct types of pragmatic inferences, and that each theoretical-pragmatic characterization of a certain type of inference corresponds to one and only one cognitive model of processing in the mind. We review the foundations of these assumptions and we problematize them based on (i) a conceptual argument that notions such as ‘implicature’ and ‘irony’ are originally meant as relations between propositions rather than types of inferences, and (ii) on recent experimental evidence which suggests that whether mentalizing is employed in pragmatic processing or not is not a function of the type of pragmatic relation, but rather it depends on situation-specific considerations and characteristics of the interlocutor, such as age and neurotype. These considerations call for a new understanding of the role of experimental evidence in the evaluation of pragmatic theories.

This article is part of the theme issue ‘At the heart of human communication: new views on the complex relationship between pragmatics and Theory of Mind’.

## 1. Introduction

The basic subject matter of pragmatics is how a given interpreter understands a linguistic string (spoken, signed or written) at a given time and context; the corresponding challenge, albeit somewhat a less investigated one, is to understand how the speaker selects the linguistic string that fits in with their conversational purposes. The output of interpretation is almost always represented as one (or several) linguistic strings, the meaning of which is put forward by the theoretician as corresponding to what the interpreter is supposed to have understood from the utterance. This is, basically, Grice’s speaker’s meaning [1].

The relationship between the linguistic meaning of the utterance and the theoretician’s representation of speaker’s meaning is usually given a particular characterization. The way such a characterization, in turn, is formulated typically appeals to the attribution of mental states to the speaker—either because it is taken to play a central role in the derivation of the speaker’s meaning or because not positing such a central role is taken as an exception which should be backed by a strong argument and usually leads to the delineation of a particular class of pragmatic meanings (think of ‘default’ or so-called ‘grammatical implicatures’ or conventionalized speech acts).

Contemporary pragmatic research routinely relies on empirical methods from experimental psychology and concepts from cognitive science to model how the comprehension of speaker meaning unfolds in the mind, as well as to gather evidence for or against such models. Experimental approaches to pragmatics are all the more valuable as more and more pragmatic theories make nuanced predictions about utterance interpretation that are not easy to check by the theorists’ own intuitions, and speculate about the underlying psychological processes (see [2–4] among others).

In the next section, however, we argue that the current epistemological foundations of experimental pragmatics rest on a long-standing potential conflation between the theoretical analyst’s representation of outputs of the interpretative processes and the cognitive mechanisms to which these representations are taken to correspond. In §3, we will claim that not keeping theoretical analyses separate from cognitive models often leads to the unscrutinized adoption of a one-to-one mapping between theoretical characterizations of pragmatic relations and cognitive models of pragmatic processing. To make our point, in §4, we will briefly review empirical work on implicature and irony that demonstrates the diversity in the cognitive models that listeners instantiate in drawing pragmatic interpretations; we will specifically focus on how mentalizing is involved, or not, in processing implicature and irony, because the role of mentalizing is often critical for theory building. We will highlight that existing experimental evidence demonstrates support for different cognitive models of processing, some of which do not employ mentalizing. By way of conclusion, in §5, we consider how clearing the epistemological conflations alluded to in the foregoing may shed new light on longstanding theoretical debates and make avenues for empirical research available. At the beginning, as often in pragmatics, there is Grice.

## 2. Typologies of pragmatic relationships rather than of pragmatic phenomena

There is no doubt that contemporary pragmatics takes its root in Grice’s [1] demonstration that most linguistic utterances can be reconstructed as instances of rational behaviour by an agent—a speaker, an author or a signer— who is seeking to transmit a message to another agent—a listener or reader or receiver—in a fully overt fashion. This message is defined as the content *q* of a mental state (a belief, a desire or an intention) of the listener; the speaker’s behaviour is then described as being compatible with the intention that the listener realizes that the speaker has the intention to cause this mental state with the content *q* in the hearer. We are aware that, at this stage, most readers are probably preparing to scroll down if not to switch to another article, because Grice’s tour de force has been discussed countless times, from introductory textbooks to intricate exegetical works. But bear with us: two crucial aspects of this foundational model have been overlooked over the years, implicitly leading to an unproblematized conflation of levels of theoretical analysis and cognitive models of processing which are tested through experimental studies.

First, Grice’s reconstruction tells us little about the speaker’s actual intentions, and even less so about how the listener processes the speaker’s utterance. In fact, Grice remains explicitly agnostic as to how the listener represents the meaning of the speaker’s utterance. As linguists, psychologists or philosophers, what we do, then, is to represent this meaning by a linguistic string, one whose most straightforward interpretation corresponds to a message it would be rational of the speaker to intend to get across in an overt way. To repeat, at this level of analysis, we do nothing more than represent the speaker’s behaviour with two linguistic strings: *p*, what we posit to be the conventional, linguistic meaning of the utterance and *q*, what corresponds to a plausible interpretation of it in a communicative context. For the rest of this article, we will stick to the following notational convention:

For any utterance, let *p* stand for the linguistic representation of the conventional meaning associated with the utterance and let *q* stand for the linguistic representation of the posited interpretation of this utterance in context.

Let us now move to the second, crucial aspect of Grice’s reconstruction: it applies to absolutely any pairing of *p* and *q*. In some cases, *p* and *q* may be substantially different (think of metaphor or irony), but in some other cases, *p* and *q* may be very close, if not identical. (Recall that the primary objective of *Meaning* [1] was to provide a rational reconstruction of conventional meaning.) Reconstructing speakers’ linguistic behaviours in terms of overt intentions to communicate a message thus holds for any type of relationship between *p* and *q*.

A rather natural move, which started from Grice’s *Logic and Conversation* [5], but which also kept replaying in many individual intellectual trajectories over the last 50 years or so, has been to move towards a classification of different relationships between *p* and *q*. Let us illustrate this with two phenomena that have probably received the largest share of attention in experimental pragmatics: quantity implicatures and irony.

Starting with the former, take the linguistic string in (1), as uttered in a conversation about the present special issue by a speaker, who can be expected, by the theorist, to be cooperative and knowledgeable about the relevant state of affairs. Typically, a pragmatic theorist would reconstruct (2) as the meaning that a putative addressee would derive from (1).

(1) Some authors used ChatGPT to write their paper. [*p*, utterance]
(2) Some, but not all authors used ChatGPT to write their paper. [*q*, speaker’s meaning]
(3) All authors used ChatGPT to write their paper.
(4) Some, but not all authors used ChatGPT to write their paper. [*p*, utterance]

The relationship between (1) and (2) is standardly called a quantity (or scalar) implicature, because (2) corresponds to the negation of the informationally stronger alternative (3), a linguistic string which (semantically) entails (2). But let us emphasize—however, trite this may seem—that there is nothing in the structure of (2) itself that makes it a quantity implicature. Rather, it is the relationship between (1) and (2) that is characterized as an implicature. In fact, if it were (4) that was uttered instead of (1), the speaker’s meaning reconstructed by the theorist would still be (2), except that this time, the relationship between the linguistic string and the reconstructed meaning would be one of identity, everything else being equal and not of implicature.

Turning to irony, imagine (5) being uttered by a teenager to their parent, rolling their eyes and with a disapproving facial expression. Typically, the pragmatic theorist would reconstruct the meaning that a putative addressee would derive from (5) as (6).

(5) Nice T-shirt. [*p*, utterance]
(6) This is a lousy T-shirt. [*q*, speaker’s meaning]
(7) This is a nice T-shirt.
(8) This is a lousy T-shirt. [*p*, utterance]

When assigned the speaker meaning in (6), an utterance of (5) is routinely called sarcasm, in great part because (6) is contradictory to (7), which is the literal representation of the linguistic meaning of (5). But again, there is nothing necessary in the structure of (5) that makes it an instance of sarcasm.^1^ In reality, it is the relationship between (5) and (6) that is characterized as such. And again, if (8) was uttered instead of (5), the theoretician would characterize the relationship between the uttered linguistic string (8) and the speaker’s meaning in (6) as an instance of identity.

While notions such as ‘implicature’ and ‘irony’, but also ‘metaphor’ or ‘indirect speech act’, merely refer to a relationship between two linguistic strings, it is a common short-cut in pragmatics to call the reconstructed speaker meanings themselves as ‘implicature’ in (2) or as ‘sarcasm’ in (6). As a result, the field often loses sight of the fact that, essentially, utterance interpretation is a coordination problem that has been modelled as a relationship between two linguistic strings, and it is these relationships that give rise to typologies of what can be roughly called ‘figures speech’ or ‘types of implicatures’.

Clearly, such typologies are situated at a level of theoretical linguistics or philosophy of language and are not concerned about the cognitive processes involved when humans produce and understand language. In other words, this way of doing pragmatics has to remain concerned with relationships between sentences uttered in a specific context and their posited speaker meanings, as a function of the context and of the structural properties of the linguistic meanings. However, the reification of structural relations between utterance meanings and posited speaker meanings into a typology of figures of speech easily leads to a double epistemological confusion. The first, which is less our concern here, is to take it for granted that the linguistic string *q* used to model the posited speaker meaning actually matches a cognitive representation the addressee forms of what is communicated by the speaker’s utterance (for a case in point, see [8,9], who argue that the *q* derived from utterances such as *p* in (1) above may well be distinct from the quantity implicature in (2) above). The second is to assume that whenever the same kind of structural relation holds between linguistic representations of the utterance meaning *p* and of the speaker meaning *q*, it should map on a single cognitive processing. There is no question that terms like ‘metaphor’, ‘implicature’, ‘irony’ and ‘indirect speech act’ can be perfectly valid explanatory categories conceptually. Indeed, the tables of contents of textbooks and handbooks in theoretical, but also experimental or clinical pragmatics, are usually shaped in terms of figures of such, with different chapters devoted to implicatures, metaphors and irony (e.g. [2,4,10–13]). However, these categories should not be associated—not without independent empirical evidence for distinct behavioural or neural signature—with specific cognitive processes involved in utterance interpretation.

## 3. From pragmatic to cognitive and experimental models

The step from rational reconstructions to cognitive explanations has been further prompted by the fact that in the classic Gricean framework rationally reconstructing speaker meanings such as (2) or (6) relies on different hierarchization of conversational maxims: e.g. quantity prioritized over quality in (2) and relation over quality in (6). Now, maxims do not correspond to cognitive mechanisms, but only to additional premises required for rationally reconstructing the speaker’s behaviour; this is why maxims can, and have been, couched in many ways (indicatively [7,13–15]). Yet, talk of maxims usually enters pragmatic theory with an agentive vocabulary, as something that the speaker actively ‘exploits’, ‘flouts’ or ‘violates’, hence, as the content of a communicative intention to be grasped by the addressee. Accordingly, when typologies of pragmatic phenomena are transferred onto the level of cognitive explanations, they often translate as different types of communicative intentions the addressee needs to attribute to the speaker to accurately grasp the meaning of their utterance.

Perhaps the earliest explicit instance of the transposition of rationally reconstructing speaker’s behaviours into a cognitive model of utterance interpretation is Strawson’s [16] account of non-conventional speech acts, framed in terms of attribution of multilayered communicative intentions. Other models followed, gradually becoming more explicit in their ambition to provide a model of cognitive processing of pragmatic interpretation, culminating in relevance theory [17,18]. All such models took for granted that the linguistic strings *q* they included as representations of the derived speaker meanings corresponded to the actual final product of the interpretation process—at the cognitive level, that is. They also presupposed that one type (of reconstructed) relationship between the utterance meaning *p* and the speaker’s *q*—implicature, metaphor, sarcasm, irony, etc.—ought to map onto a single cognitive process.

For instance, while for relevance theorists like Sperber & Wilson [18] or Carston [19], any kind of pragmatic interpretation involved a unitary mechanism, Recanati [20] distinguished between primary, or local processes and secondary, or more global inferences (see [21,22] for a detailed discussion). Interestingly, the dividing line was usually in the extent that mentalizing should be involved. Thus, both relevance theorists and Recanati modelled irony in terms of (broadly Gricean) attribution of communicative intentions, but for Recanati, no such attribution was involved in the causal interpretation of the two conjuncts of (9), because the latter, unlike the former, is a primary pragmatic process.

(9) Peter left Mary and she started to drink.

These models of pragmatics provided impetus for contemporary experimental approaches, but remained in fact relatively agnostic as to how they should be empirically tested with the methods of experimental, developmental or clinical psychology. The scholars of pragmatics who situated themselves at the level of (cognitive) theorizing mostly recurred to a notion of multilayered attribution of mental states that retained much of its original formulation in Gricean reconstruction, sometimes coupling it with rather loosely defined concepts such as accessibility or salience [6,18,20].

As just discussed, the shift from rational reconstructions or cognitively inspired theories to cognitive models has rarely, if ever, been explicit. And the experimental approach that followed from 2000 onwards retained both the idea of a typology of pragmatic processes and the central role assigned to complex mental state attribution. From a psycholinguistic point of view, psychological processes that are involved in utterance interpretation range from perceptual to attentional to representational: they include activation, retrieval, inhibition, flexibility, short-term and working memory, creativity, emotion regulation, but also representation of additional information that is required for the understanding of the linguistic utterance, important among which is the representation of the interlocutor knowledge. Psycholinguistic explanations readily acknowledge the complexity inherent in such processes—and devote much attention to carefully teasing apart their differential contribution to utterance interpretation. Experimental pragmatics, however, often operates on the assumption that mentalizing should have a straightforward translation from theories to cognitive models, despite the long-standing difficulties in operationalizing, delineating and reliably testing ‘Theory of Mind’ in cognitive, clinical and developmental research (e.g. [23–27]).

But let us accept, for our current purposes, a loosely defined ‘mentalizing’ construct [25], which may correspond to any kind of understanding of the interlocutor’s perspective or cognitive representation. The point we will now illustrate using the same two phenomena as above—quantity implicatures and irony—is that there should be no one-to-one mapping of typologies of pragmatic phenomena onto cognitive models delineated by their relationship to mentalizing. There is nothing wrong, of course, with typologies of pragmatic outputs inspiring cognitive models, to which we most certainly owe the experimental turn in semantics and pragmatics that has characterized the first quarter of this century. In fact, we would argue, together with everyone else in the field of experimental pragmatics, that studying the processes involved in the production and comprehension of pragmatics at the behavioural and neural level can be highly informative for pragmatic theory, as well as for psycholinguistics and cognitive science. What is wrong is to assume that because one chooses a certain characterization of the relationship between *p* and *q* at the theoretical level (say, as a quantity implicature or irony), there only needs to be one and only one model of pragmatic processing that speakers and hearers instantiate to reach *q* from *p* (see also [22,28,29]).

## 4. Mentalizing and its role in cognitive models of pragmatic processing

### (a) Case-study I: quantity implicatures

Consider (1)–(4) above as well as (10)–(13) below:

(10) He picked the basket with pears. [*p*, utterance]
(11) He picked the basket with pears and nothing else. [*q*, speaker’s meaning]
(12) He picked the basket with pears and bananas.
(13) He picked the basket with pears and nothing else. [*p*, utterance]

In both sets of examples, the relation between *p* and *q* is that of a quantity implicature: (12), which has a critical role in the characterization of (11) as implicature, conveys more information or is informationally stronger than (10). The role of pragmatic theories is to provide an account of the origins and characteristic properties of interpretations that arise through quantity implicature. Broadly, in Gricean terms, a theory of implicature would propose that the interpretation arises due to interpersonal considerations of cooperativity which include truthfulness, informativeness and relevance as guiding principles of the conversational interactions of rational agents. From that theory, it is possible to derive a step-by-step narrative account of the reconstruction of the relationship between *p* and *q* (see [30]):

(i) Speaker has said *p.*
(ii) There is a more informative alternative utterance which is relevant in the conversation at hand, *q*, which the speaker did not say
(iii) A speaker who is cooperative and informative, would have said *q*, as long as *q* is relevant.
(iv) Therefore by saying *p* the speaker is implicating that she does not know if *q* is true or not.
(v) A speaker who is knowledgeable would know whether *q* is true or not.
(vi) By saying *p* the speaker is also implicating that *q* is not true.

The relation between step (i) and step (iv) is typically considered a weak implicature, while the relation between steps (i) and (vi) is the so-called strong quantity implicature [31]. Importantly, to derive the second implicature, the one that intuitively is available when one says *p*, the listener must consider the speaker’s epistemic state, thereby making mentalizing, or making inferences about a speaker’s intentions as well as about their knowledge state, an integral part of pragmatics.

Some pragmatic theories kept close to Gricean derivation (consider Horn’s [14] re-organization of Grice’s four maxims into two or Levinson’s [13] proposal for three maxims); in all such theories, that can be dubbed *interpersonal*, for an implicature to be generated, a listener would reason along the steps starting from (i) and concluding at (vi) (see also [31]). Other accounts of quantity implicatures, however, can be called *form-based*, as they depart more boldly from Grice, in several respects, including the requirement that the alternatives to *q* be propositions rather than sub-propositional constituents, and that the steps (i) to (vi) do not explain the intuitive implicature derived by the listener in cases where the critical expression is embedded within quantifiers and other logical operators (see [7]). Within the form-based group of accounts, there are also proposals that the derivation of the alternative utterance and its negation is performed by the grammar, using an operator akin to a silent ‘only’, i.e. the tokening of ‘some’ in *p* triggers the insertion of a silent ‘only’, which leads to the derivation of ‘not all’ (e.g. [32] and many more recent instantiations of grammar-based accounts of implicature).

While such various proposals disagree on how quantity implicatures are best characterized, they all argue that one single characterization of the relation between *p* and *q* is the correct one. For interpersonal theories the condition that the addressee of *p* is knowledgeable about what the speaker of *p* knows (and also that the speaker of *p* is cooperative, and relevant, given the contextual goals) is necessary for an implicature to arise. For form-based theories and their models, this is not required. At best, if it turns out that the speaker was not knowledgeable (or cooperative or relevant) then the implicature will be cancelled [7].^2^

Accordingly, what also unites interpersonal and form-based cognitive models of implicature processing is that they both commit to there being one cognitive model that describes the psychologically real process that takes place in the listener’s mind. Interpersonal accounts are typically associated with a cognitive model where the listener considers the informative proposition that was not said *q*, only if the listener believes that the speaker would be in position to say *q*. By contrast, form-based cognitive models place mentalizing about what the speaker knows as the last stage of utterance interpretation. Put simply:

(a) Interpersonal models: mentalizing about speaker’s intentions and knowledge, then consideration of more informative alternatives speaker has not said
(b) Form-based models: consideration of more informative alternatives speaker has not said, then mentalizing (if at all)

The one-to-one assumption that a pragmatic theory should map onto a single cognitive model and vice versa, is so pervasive that it is not even stated and simply taken for a fact. For instance, in most research on whether mentalizing is implicated in implicature acquisition and processing, there is no other hypothesis entertained bar the binary one: evidence that mentalizing is employed supports an interpersonal model and evidence that it is not supports a form-based one ([30,34–37] among others).

**Figure 1.**
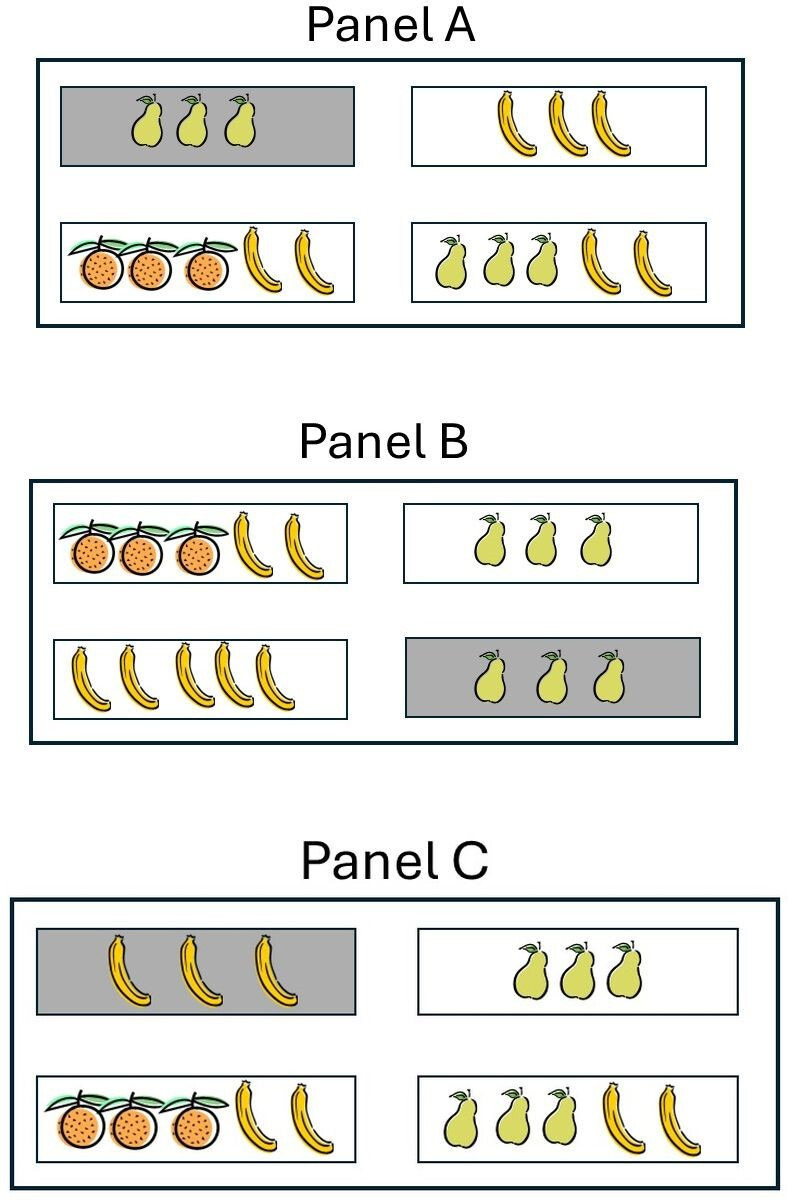
Three panels (Panel A, Panel B and Panel C) with indicative displays from a listener’s perspective from Katsos *et al*. [38] study, for the instructions ‘Pick the card with pears’. The card with a shaded background is visible to the listener but not the speaker.

However common, this is exactly the assumption that recent experimental research can challenge. Katsos, Wilson and colleagues have recently reported evidence that both cognitive models can be employed, depending on the listener and on the conversational setting. They employed simple schematic situations with cards depicting certain items (fruit), with a speaker who asks for a card but can only see three out of four cards, with the remaining card being visible only to the listener [38–40]. In critical conditions, the card that a listener will reach for if they engage with mentalizing before engaging with reasoning about informative alternatives differs compared with if they apply informativeness without mentalizing (see figure 1, Panel A).

Starting with child language acquisition, Wilson *et al*. [41] looked at 5−7 year old English-speaking children who could all pass a first-order false-belief task (Sally-Anne task) as a precondition of participation. To further assess participants’ ability to mentalize about the speaker’s knowledge at a level that is necessary for success in this study, there was also a mentalizing condition within the main experiment that required plain visual perspective-taking (e.g. there were two identical target cards, one of which was hidden from the speaker, rendering only the one that was seen by both speaker and listener as the target; see figure 1, Panel B). Moreover, there was a condition where the selection of the correct card depended on participants’ ability with informativeness, regardless of mentalizing (because whether the participant applied informativeness having first considered what the speaker knows or without regard to it, led to the same response; see figure 1, Panel C). While children performed very well with informativeness on its own (in conditions with displays such as in figure 1, Panel C), and reasonably well with perspective taking on its own (in conditions with displays such as in figure 1, Panel B), in the critical condition (see figure 1, Panel A) in the overwhelming majority of cases, children chose the card that was consistent with informativeness, but without mentalizing (which, in cases like those depicted in figure 1, Panel A would correspond to giving the speaker the card with pears only).^3^ There also was a group of adults in the study, who predominantly selected the card consistent with mentalizing taking place before informativeness is applied.

These results are compatible with two cognitive models where, for most developing users of language, reasoning about informativeness applies without mentalizing, while for mature users of language, informativeness applies only after mentalizing has taken place. However, it might be argued that this conclusion does not necessarily raise a substantial challenge to the one-to-one mapping between pragmatic theories and cognitive models, because the specifics of the model instantiated is contingent on the language users’ experience and maturation. For example, when investigating children’s ability with metaphor, Lecce *et al*. [42] found that 9-year-old children were able to understand physical-perceptual metaphors, but were having challenges with psychological metaphors, which–for several reasons–have higher mentalizing requirements (see [42]). In their study, children employed their pragmatic abilities to interpret psychological-metaphor utterances as metaphorical rather than literal, but they did so without mentalizing at the appropriate level, giving the utterances interpretations that were based on a physical-perceptual dimension of analogy rather than a psychological one. In a similar vein, one could ask if the children in Wilson *et al*. [41] were interpreting the critical utterances without mentalizing because of the high load of mentalizing, a strategy that they will abandon once their ability to mentalize develops further. However, whereas in the study by Lecce *et al*. [42] the mentalizing load between the two types of metaphor is different, in the study by Wilson *et al*. [41] the mentalizing load for taking the speaker’s perspective is the same between the critical utterance (figure 1, Panel A) and the control mentalizing condition (figure 1,Panel B). Moreover, every child that was selected to complete the task had independently passed a first-order Theory of Mind task (the Sally-Anne task), which could easily be argued to have an even more complex linguistic structure than the critical task. Therefore, it seems unlikely that the critical condition’s demands on mentalizing was what was driving some of the children to resort to an interpretative process without mentalizing. Instead, in the Wilson *et al*. [41] study, some children who patently have the ability to mentalize to the extent required in the critical condition, do not do so. The reasons for this will be discussed further, after we summarize similar findings from adult participants, for whom explanations that capitalize on not-yet-fully-mature developmental stages are even less likely to apply.

In further investigation, Katsos *et al*. [38] looked into more detail in English-speaking adults and found that when presented with displays such as in figure 1 Panel A, a majority of adult respondents selected the card consistent with mentalizing in line with the findings for adults by Wilson *et al*. [41]. However, Katsos *et al*. [38] also found, across two studies, that there were consistently 20–25% of responses where adults selected the card consistent with the lack of mentalizing. This finding raises substantial challenges for the one-to-one mapping assumption, because evidence for two cognitive models is now found within the same participants, and indeed, within an adult sample. That is, one and the same adult relied most of the time on mentalizing but on some occasions interpreted an utterance without. As with the experiments reported by Wilson and colleagues, there was also a mentalizing control condition, which adults passed with near-ceiling performance. The contrast between adults’ success with the mentalizing control condition on the one hand (see figure 1, Panel B) and the condition where mentalizing of the same complexity was interacting with pragmatic inference (see figure 1, Panel A; 93 versus 75%, respectively, in Experiment 1B) highlights that mentalizing load is unlikely to be the explanation for the evidence of two cognitive models. Moreover, with the same experimental paradigm, Katsos *et al*. [38] found that autistic adult participants were not employing mentalizing as much as their neurotypical peers not only for the critical condition, where mentalizing and informativeness would clash, but also across the other conditions of the experiment.

In further ongoing research, Katsos *et al*. increased the interactiveness of the participants’ experience, with the speaker issuing instructions in real time through a chat box, and with simple additions of politeness markers and markers of interactional history in the instructions that the speaker issued (such as changing ‘pick the card with pears’ to ‘this time, could you pick the card with pears please’). This created a higher interaction paradigm where the participants are now more accountable for their actions to an interactional partner who is co-present temporally. In the pilot version of the experiment (reported in [39]), virtually all adult participants selected the card with pears in the critical condition, with no responses based on a cognitive model where informativeness would take place without or prior to mentalizing.

In summary, the evidence surveyed in the foregoing supports both interpersonal and form-based models of pragmatic processing of one and the same pragmatic relation, that of quantity implicature. In turn, the above suggests that there is a one-to-many mapping between the theorist’s notion of quantity implicature and the cognitive models through which it is instantiated. The likelihood of which model is used is predicted by the age of the listener (children or adults), the neurotype (neurodivergent or neurotypical), as well as the conversational situation (high or low interactivity). In addition, there is evidence in favour of both models being used even within what would be considered a relatively homogeneous group of adult neurotypical listeners, highlighting that the diversity of cognitive models instantiated in pragmatic processing is pervasive. We will explore further the implications of these findings in the §5.

### (b) Case-study II: irony and mentalizing

Irony is probably the clearest case where mentalizing seems inevitable [43], and many studies have reported that mentalizing is involved in irony comprehension (e.g. [44–46]). Once you know that there is no way the speaker of (5) may like your t-shirt, what it takes, at the core, to understand this utterance as a sarcastic remark and not as a (compassionate) lie is the ability to get that the speaker clearly knows that you know that they do not actually believe that your t-shirt is nice. This kind of complex mentalizing is also involved in deceiving other people, and it is not surprising that children begin to grasp the distinction between jokes and lies at the same age as their lies become efficient [47]. Keeping track of the mental states of other people in conversation may prove difficult for autistic individuals, who also often struggle with irony [48].^4^

All this, however, does not mean that every time an utterance is interpreted as sarcastic—or, for that matter, a sarcastic interpretation is considered and ruled out—hearers instantiate a cognitive model where mentalizing plays a central role. This is particularly true in situations where a shallower processing may be favoured. In Deliens *et al*. [51] participants had to decide, from a third-person perspective, whether the addressee of a message left on their voice mail would have interpreted it as ironic or not (see also [52]). In some trials, the preceding context made the ironic interpretation unambiguously salient to both study participants and the addressee, as in the following example:

(14) [Context:] Anaïs is going on vacation to Barcelona. Her friend Clemence would like to visit the city soon too and asks Anaïs to tell her what she thinks of her hotel. Therefore Anaïs, once there, sends Clemence a post card saying: ‘Dear Clemence, you will love Barcelona as long as you do not stay at the hotel we are in: it is dodgy, ugly and dirty! That aside, it is all sun and parties!’ A few days later, Anaïs is back from her holidays and wants to call her friend to tell her about her trip. As she reaches [Clemence’s] mail, she leaves a message:

Hi Clemence, I’m back! I need to tell you about our hotel: a small and charming place and impeccably clean.

In other trials, however, the participants were provided with information that was not available to the utterance addressee, but strongly compatible with an ironic interpretation from their, participants’ point of view. One such trial is illustrated in (15): this time, the ironic interpretation of the message on the voice mail made sense from the participant’s egocentric perspective, but not from that of the addressee.

(15) [Context:] Anaïs has always been a good student. When her friend, David, sees her on her way to her final exam, he tells her once again: ‘Don’t worry, you always know the material from A to Z. You will nail the final exam.’ Actually, as never before, Anaïs has a black-out and can’t even understand most of the questions. When David goes back home in the afternoon, he finds a message saying:

Hey David, you are right, there was no reason to be anxious, the exam went smoothly.

Interestingly, when participants were placed under time pressure in such trials, they did not engage in allocentric perspective-taking, and tended to judge the utterance ironic, even in cases where from the addressee’s perspective it was clearly not. This result shows that perspective-taking in assessing irony is cognitively costly and is not automatically used. In some trials of Deliens *et al*. [51], the target message was also produced with a salient intonation; in these trials, hurried participants’ reaction time indicated that they directly accessed the ironic interpretation, without considering the addressee’s perspective.

Deliens*et al*. [53] designed an act-out task, where participants had to decide which of two items, e.g. a physics or a chemistry book, an adult wanted, based on exchanges like utterances such as (16).

(16) Adult 1: Here is a physics book and here is a chemistry book. Would you like the physics book as a gift, now?

Adult 2: Yes, you know how much I like physics! [Target sentence]

These target utterances could be produced with marked or neutral intonation, ironic or not, combined or not with a marked facial expression, again, ironic or not; furthermore, in some conditions, a preceding context made Adult 2’s preferences clear (e.g. he did not like or love physics). Such a context significantly increased the accuracy of irony detection, but was also associated with longer reaction times—confirming the cost of taking the speaker’s mental states into account. Interestingly, marked intonation did not have a beneficial effect on detection of irony and marked facial expression even had a negative one, but both were associated with shorter reaction times ([53] Exp. 2 and 3). That is, within the same irony comprehension task, participants used different strategies, sometimes dispensing with cognitively costly mindreading by relying on more directly accessible, but less reliable, cues.

It is worth noting, in this respect, that in an independent force-choice experiment, participants did reliably discriminate between the same ironic and not ironic target sentences as in Deliens *et al.* ([53] Exp. 2 and 3), when these were presented in isolation and based solely on intonation or facial expression ([53] Exp. 1). This demonstrates a discrepancy between discrimination, meta-linguistic tasks and act-out paradigms, where different cues co-occurred in combination and a decision on the meaning communicated by the speaker of the target sentence had to be made. In the same vein, in binary, force-choice tasks autistic individuals may accurately classify utterances with a marked intonation as sarcastic [54,55]. However, in paradigms where salient intonation cannot serve as a binary cue, and the speaker’s perspective ought to be taken into account, autistic individuals perform at chance on irony detection [56].

That utterances are processed as ironic or not based on multiple cues is, of course, not surprising [57]. Our point is simply that there is no such thing as ‘ironic processing’, meaning that there is not one single way of grasping irony: when, as theorists, we speak about irony we merely characterize a relationship between a linguistic meaning *p* and what we suppose to be the derived meaning *q* that is incompatible with *p*. In some situations, hearers will not need to take the speaker’s perspective to derive *q*, and in some others, simply understanding that *p* is not the speaker’s meaning will be sufficient (think, for instance, of situations in which a certain tone of voice suffices for you to decide that a speaker is being sarcastic and it is enough for you that they do not mean what they say, without you trying to understand what exactly they are trying to convey). Again, what kind of cognitive model will be instantiated in the interpreter’s mind depends on their cognitive profile, as well as on situational characteristics and demands.

## 5. Discussion

In this contribution, we have made several proposals. First, that in standard Gricean pragmatics, implicature, irony and other pragmatic phenomena are relations between propositions that theorists propose and aim to characterize with theory-internal tools; they are not intended to be types of inferences with cognitive reality. When, as Gricean theorists, we write of listeners ‘making’ or ‘generating’ or ‘deriving’ an implicature (or any other pragmatic phenomenon), this is a short-hand rather than a commitment to there being a single distinct cognitive process in the mind which corresponds to an implicature. While there may come to light evidence that the mind and the brain engage in some kind of process that is unique and dedicated to the processing of implicature, irony and so on, we are not aware of such evidence yet. What psychological science gives us to date is processes of meaning retrieval, activation, inhibition and so on and so forth, and it is important to think of how we would understand implicature at the cognitive level in these terms. Second, we problematized the widespread, commonly held, but virtually always implicit assumption that only one cognitive model of pragmatic processing may instantiate a certain type of pragmatic relation (which the present authors themselves have certainly held themselves, in the past). Recent evidence from implicature and irony suggests a one-to-many mapping, whereby different cognitive models may be instantiated in different speakers or in the same speaker across different situations. Where does this leave us?

A first question is that of the epistemological status of individual differences. One may argue that we could be content with providing a cognitive model that describes the typical or the majority listener rather than capturing the diversity observed in the evidence reviewed above. For instance, there are substantial differences in people’s ability to engage with visual perspective-taking, as well as the ways in which they engage with it (see [58] for a review). Would it not be plausible to argue that evidence of differences on how implicature or irony is processed might reflect fundamental individual differences in basic perspective-taking abilities? This then might offer a way out from confronting the implications of diversity, by arguing that pragmatic theories reflect how the majority of speakers/hearers represent the relation between *p* and *q*; hence, they can be used to make predictions about the majority of responses allowing for exceptions or differences in minority cases.

We think this is not a productive line of argument for two reasons. First, it introduces assumptions about typicality and majority among groups that would otherwise be considered equally central to pragmatic competence as neurotypical adult native speakers. Second, in line with the results in the visual perspective-taking literature, the diversity in pragmatic processing is modulated not only by differences among individuals engaging in the same task, but it is also a function of the conversational situation, giving rise to task-dependent diversity of processing. The existence of more than one cognitive model of pragmatics is therefore something that has to be addressed when it comes to experimental pragmatics.

A handful of theoretical papers do challenge the one-to-one mapping between theoretical characterisations of pragmatic phenomena and cognitive processes. An early proposal by Sperber [29] argued that the complexity of cognitive processes involved in interpretation is a function of the communicative situation and maturation of the addressee’s cognitive capacities, rather than the pragmatic phenomenon itself, using relevance implicature as a case study. He proposed a hierarchy of cognitive interpretative strategies based on increasingly complex meta-representations of the interlocutors’ beliefs and intentions. In this line of work, mentalizing is axiomatically part of the interpretative model, and what is at stake is the degree of complexity. However, Kissine [21,22,59] draws from evidence from language acquisition, and from language and communication in autistic children and adults to make a claim that is similar in its structure, except that it is proposed that models without mentalizing are available to interlocutors as well. In a similar vein, Katsos & Andrés-Roqueta [28] also propose that interlocutors may process pragmatics with or without mentalizing, following a review of evidence from autistic language processing.

Katsos & Andrés-Roqueta [28] put forward a situation-based view on pragmatics and mentalizing, whereby the critical distinction that modulates whether the latter will be employed is one between communicative situations. Drawing on work on alignment and grounding [60–62], they propose that conversational situations differ with respect to how likely the interlocutors are to be mentally aligned. Indicatively, in situations where there is a successful history of communication, physical and temporal co-presence of the speaker and the listener, common goals and joint attention in the task at hand, interlocutors are more likely to assume they are engaged in an activity where their mental states are aligned. In such situations, actual mentalizing is less likely to occur, and listeners are likely to be most frugal in how to represent the speaker’s intentions and beliefs. This leads listeners to assume that their partners’ mental world—their knowledge, preferences and intentions regarding the communicative situation—are identical to their own (i.e. the listeners’). In other situations, where there are cues that the speaker and the listener might not share common ground, might have competing goals or different abilities to express themselves in conventional ways, the listener will be more likely to employ actual mentalizing to represent the speaker’s knowledge state and intentions as accurately as possible. Katsos & Andrés-Roqueta [28] talk about two different interpretative strategies, social pragmatics and linguistic pragmatics, which correspond to situations where the interlocutors are more and less likely respectively to consider that their mental states are aligned. The question of diversity of interpretative strategies then becomes a distinction about the nature of communicative situations, and of interlocutors’ ability to correctly monitor which kind of communicative situation they are in. Briefly returning to the evidence from informativeness reviewed above, one will recall that children and adults who applied informativeness without mentalizing in the critical condition were perfectly able to mentalize at the appropriate degree to succeed with the task (as shown by passing first-order false-belief tasks, for example, as well as passing the mentalizing control condition within the main experiment). Therefore, using mentalizing or not was not a property of the pragmatic phenomenon itself (or else all participants would have mentalized or not), nor of the individuals’ competence *per se* (because the speakers were able to mentalize at the required level), but rather of the individual’s ability to monitor that they were in a situation where mentalizing was needed.

Kissine [22] argues that, from a processing point of view, pragmatics is a meta-cognitive process, which both determines the target of interpretation (getting exactly what speakers have on their mind, validating a salient expectation, choosing between two options, and so on and so forth) and guides interpretation towards it. Under this view, pragmatic processing is akin to solving problems such as passing an exam, where one needs to understand the goal of the process (for example, repetition of learned material or critical interpretation) and monitor the achievement of this goal (for instance, using memory, contextual cues and associative processes). And just as there are two ways to fail an exam—by mistaking the goal or by choosing the correct goal, yet failing to reach it—there are several ways pragmatics may fail us: we may not understand that someone is ironic or we may understand that an ironic interpretation is to be reached, but we fail to infer the correct content of the message the speaker is trying to get across. Importantly, though, both aspects of pragmatic processing, that of determining the goal of interpretation and of the control loop that monitors its achievement, rely on a variety of contextual factors, that may, but need not, involve mentalizing.

The combination of the proposals by Kissine [22] and Katsos & Andrés-Roqueta [28] is that the determination of which interpretative strategy will be employed and which cognitive model will be instantiated is a function of communicative situations and of individuals’ developmental stage or neurotype. Unlike other work [18,29] whereby all the possible strategies and models include (simpler or more complex) mentalizing, here we are confronted with an argument and evidence that one of the possible models does not involve mentalizing at all. Pragmatic interpretation remains, in principle, an exercise on behalf of the listener to infer the intention the speaker had by uttering *p*. It is just that, somewhat paradoxically, there are circumstances when this might happen without mentalizing about the speaker’s intentions or knowledge. One way to understand this would be to assume that sometimes lexical, syntactic or acoustic characteristics of the utterance give rise to an interpretation that is sufficient from the interpreter’s point of view (see also [63,64] for related positions). Another would be that in some circumstances the speaker erroneously considers what the common ground between themselves and their interlocutor is. They might, for instance, consider that the common ground is identical to their egocentrically based beliefs, which would be indistinguishable from not mentalizing at all [52,65,66]. It is theoretically possible that what appears to be egocentric processing is, in fact, the outcome of mentalizing which has failed to deliver what actually is in the speaker’s perspective. In the case of quantity implicatures, for instance, this would lead the listener to construct a common ground that contains plausible referents identical to those of an egocentric listener who would not be engaging in mentalizing and constructing a common ground. Further research is needed to outline testable predictions to clearly tease apart models according to which pragmatics always involves mentalizing, albeit sometimes returning an output that is indistinguishable from that of an egocentrically biased model (see [67], among many others) from the one we defended here which allows for pragmatics without mentalizing.

What does this mean for pragmatic theory? Traditionally, in major debates in the theoretical and experimental pragmatics, whether mentalizing is involved or not is a categorical criterion either about the pragmatic or not nature of the relation under study (think of Gricean conversational implicatures versus Chierchia’s grammatical implicatures) or, in other cases, mentalizing delineates subclasses of relations (think of Recanati’s primary and secondary pragmatic processes or of Levinson’s default versus particularized implicatures). Experimental evidence for or against the use of mentalizing has been used to draw conclusions about which theory of implicature—or irony, or metaphor, or speech acts—is on the right track. The situation-based individual-competencies view that we outlined above suggests that theories should not position themselves on this or the other side of a notional divide, but instead they should aim to predict when mentalizing will be engaged. Similarly, the role of experimentalists is to unveil the range of interpretative strategies that are available, and to point to the factors that contribute to which strategy is more likely to be employed. Having made a few first steps in this direction, the challenge for theoreticians and experimentalists alike will be to develop theories that take into account and can predict this diversity of cognitive processes, rather than theories that make one or the other cognitive process exclusively the cornerstone of pragmatics.

## Data Availability

This article has no additional data.

## Footnotes

^1^
This is not to deny, of course, that there are some linguistic strings that are more prone to an ironic interpretation than others [6]. Likewise, quantified sentences are particularly prone to giving rise to quantity implicatures [7]. The point remains that, regardless of the degree of potential conventionalisation, it is the relationship between the linguistic meaning and the posited speaker meanings that underlies these pragmatic labels.

^2^
There have been exceptions to this, though they are not critical for the point we are making here. Noveck & Sperber [33] argue that in relevance theory terms the relation between *p* and *q* may sometimes be characterized as an implicature (part of Grice’s ‘speaker meaning’) and sometimes as an explicature (part of Grice’s ‘what is said’), depending on the conversational context. However, in both cases, whether it is a relation of explicature or implicature it is one and the same interpretative principle that is at play, and this includes considerations from the context of the conversation at its core, thereby making relevance theory one of the interpersonal theories, rather than the form-based ones. In other words, the distinction between implicature and explicature is not one of different pragmatic principles being at play.

^3^
We would like to thank Ira Noveck for asking whether the choice of the card without mentalizing is due to this card being a more attractive option than the alternative card. This is something that [40] and Katsos *et al*. [38] discuss extensively with conceptual arguments and follow-on experiments, and we refer the reader to the original studies if they wish to explore this point further.

^4^
As has been long noted, beginning with Grice himself, the conversational aim of irony is not merely to mean the opposite of what is literally said, but rather to convey some kind of (most often) negative attitude towards this content [44,49,50]. It remains that on classic accounts engaging in complex mentalizing is required to detect that the speaker is being ironic.
